# Supplementary material for: Characterisation of Non-Pathogenic Premutation-Range Myotonic Dystrophy Type 2 Alleles
Source: J Clin Med. 2021 Aug 31;10(17):3934. doi: 10.3390/jcm10173934 (PMC8432210; doi:10.3390/jcm10173934)
Supplement: Supplementary file 1 [file jcm-10-03934-s001.zip › jcm-1311152-supplementary.pdf]

Supplementary Table S1: Inheritance of interrupted alleles – measured in lengths of the complex repeat motif. **Blue bold** numbers highlight interrupted alleles which are shared between generations in each family, while **red bold** numbers indicate uninterrupted alleles. Family DM2 is identical to Family_2 in the manuscript, while Family_1 is part of an extensive family marked as DM-125. Exp= expanded allele.

Family identifier	Relationship	*CNBP1* complex motif length (bp)	
		Allele 1	Allele 2
DM-1	Father	138	**154**
DM-1	Mother	**122**	**198**
DM-1	Son	**154**	**202**
DM-2	Father	132	**134**
DM-2	Son	**134**	152
DM-2	Son	**134**	152
DM-3	Mother	138	**140**
DM-3	Daughter	124	**140**
DM-7	Mother	134	**134**
DM-7	Son	134	**134**
DM-7	Son	134	**134**
DM-8	Father	**124**	142
DM-8	Mother	**132**	**134**
DM-8	Son	**124**	**134**
DM-8	Son	**124**	**132**
DM-10	Father	**138**	140
DM-10	Mother	132	**142**
DM-10	Daughter	**138**	**142**
DM-11	Father	140	**148**
DM-11	Son	138	**148**
DM-11	Son	138	**148**
DM-13	Father	**138**	146
DM-13	Mother	134	**138**
DM-13	Son	**138**	**138**
DM-18	Mother	128	**136**
DM-18	Daughter	**136**	140
DM-18	Son	130	**136**
DM-20	Father	128	**130**
DM-20	Mother	**132**	138
DM-20	Son	**130**	**132**
DM-53	Mother	128	**138**

DM-53	Son	132	138
VKF-1	Mother	126	140
VKF-1	Son	136	140
VKF-1	Son	130	140
DM-63	Mother	138	Exp
DM-63	Son	136	138
DM-63	Daughter	136	138
DM-101	Father	132	146
DM-101	Daughter	132	142
DM-105	Mother	142	152
DM-105	Daughter	142	Exp
DM-105	Daughter	152	Exp
DM-120	Mother	132	Exp
DM-120	Daughter	128	132
DM-122	Mother	136	138
DM-122	Amniotic fluid	132	138
DM-122	Son	138	152
DM-122	Daughter	138	142
DM-125_1	Mother	130	140
DM-125_1	Son	126	140
DM-125_1	Son	126	130
DM-125_1	Daughter	126	140
DM-125_2	Grandmother	156	**186**
DM-125_2	Daughter_1	134	156
DM-125_2	Grandson_1 (Son of Daughter_1)	134	144
DM-125_2	Granddaughter_1 (Daughter of Daughter_1)	134	140
DM-125_2	Grand-granddaughter_1 (Daughter of Granddaughter_1)	134	142
DM-125_2	Granddaughter_2	146	**190**
DM-125_2	Grand-grandson_2 (Son of granddoughter_2)	140	146
DM-125_2	Daughter_3	144	156
DM-141	Mother	132	140
DM-141	Son	140	140
DM-141	Son	132	140
DM-142	Mother	134	142
DM-142	Son	132	142
DM-150	Father	138	138
DM-150	Son	134	138

DM-166	Mother	136	136
DM-166	Daughter	130	136
DM-197	Mother	142	142
DM-197	Son	140	142
DM-199	Mother	142	Exp
DM-199	Daughter	138	142
DM-206	Father	138	142
DM-206	Mother	132	138
DM-206	Daughter	138	142
DM-206	Daughter	132	138
DM-242	Mother	138	138
DM-242	Daughter_1	138	Exp
DM-242	Granddaughter (Daughter of Daughter_1)	136	138
DM-242	Son	138	142
DM-242	Daughter_2	138	142
DM-271	Mother	152	152
DM-271	Daughter	142	152

Supplementary Table S2: Intergenerational instability/stability of the uninterrupted *CNBP* alleles and 17 other microsatellite loci (*DMPK* and the loci included in the new European Standard Set (ESS) of loci, plus SE33) in selected family members. Results of the gender specific amelogenin and the rs1871922 genotypes are also shown.For the *CNBP1* locus uninterrupted alleles are shown in bold and are underlined, while "i" indicates interrupted alleles. "A/C" = heterozygous state without available phase information.

Locus	Repeat motif	Chrom. position	Family_1				Family_2						Family_3						Family_4			
			Grandmother		Index case		Mother		Index case		Son		Mother		Index case		Son		Mother		Sister of index case	
			All.1	All.2	All.1	All.2	All.1	All.2	All.1	All.2	All.1	All.2	All.1	All.2	All.1	All.2	All.1	All.2	All.1	All.2	All.1	All.2
AMEL (X/Y)	-	Xp22.1-22.3 Yp11.2	X	X	X	X	X	X	X	Y	X	Y	X	X	X	X	X	Y	X	X	X	X
TH01 (*TC11*)	[TCAT]n	11p15.5	9.3	9.3	9	9.3	8	9	8	9.3	9	9.3	8	9.3	8	9.3	6	8	6	7	6	9.1
D3S1358	TCTA [TCTG]n [TCTA]n	3p25.3	15	15	15	16	17	17	17	18	17	17	14	17	14	16	14	15	14	17	15	17
vWA	TCTA [TCTG]n [TCTA]n	12p13.31	16	17	16	17	17	17	17	17	17	18	17	18	16	17	15	16	16	18	18	18
D21S11	[TCTA]n [TCTG]n [TCTA]n TA [TCTA]n TCA [TCTA]n TCCATA [TCTA]n	21q21.1	29	30	30	32.2	28	29	29	29	29	30	28	30	28	29	28	29	29	30	29	32
D16S539	[GATA]n	16q24.1	9	12	9	13	13	14	11	14	11	13	12	12	11	12	9	12	11	11	11	14
D1S1656	[TAGA]n [TGA][TAGA][TAGG]n[TG]n	1q42	17	19.3	15.3	16	17.3	18.3	12	18.3	16	18.3	15.3	16	15	16	16	17	16	17.3	16	17.3
D19S433	AAGG [AAAG] AAGG TAGG [AAGG]n	19q12	13	13.2	15	16	14	16.2	16.2	19	14	16.2	6.2	13	13	14	13	14	14	14	14	14
SE33 (*ACTBP2*)	[AAAG]n AA [AAAG]n	6q14.2	17	26.2	17	25.2	14	24.2	24.2	31.2	30.2	31.2	19	29.2	16	29.2	19	29.2	15	26.2	15	17
D10S1248	[GGAA]n	10q26.3	13	15	12	14	14	16	15	16	14	15	14	15	14	14	14	14	15	16	15	16
D22S1045	[ATT]n ACT [ATT]n	22q12.3	15	15	11	14	11	15	11	11	11	18	16	16	16	16	16	17	15	16	11	15
D12S391	[AGAT]n GAT [AGAT]n [AGAC]n AGAT	12p13.2	17	18	17	23	16	19	18	19	18	18	20	22	17	22	17	22	19	22	22	22
D8S1179	[TCTA]n	8q23.1-23.2	12	13	13	15	13	14	13	14	13	14	14	16	14	14	13	14	13	14	14	14
D2S1338	[TGCC]n[TTCC]n	2q35	19	24	19	24	20	20	20	25	24	25	22	23	20	23	18	23	20	24	18	20
D2S441	[TCTA]n	2p14	12	14	14	14	11	15	11	15	11	15	11.3	11.3	11.3	13	11	13	11	11	10	11
D18S51	[AGAA]n	18q21.3	14	15	15	17	13	14	14	15	13	14	14	14	14	15	12	15	14	18	17	18
FGA (*FIBRA*)	[TTTC]n TTTTTTCT [CTTT]n CTCC [TTCC]n	4q28.2	19	22	23	23	21	24	20	24	21	24	22	25	22	25	22	23	23	23	23	24
DMPK	[CTG]n	19q13.3	13	13	14	Exp	12	13	5	12	11	12	11	11	5	11	5	5	13	14	5	13
CNBP1	[CCTG]n alone	3q21.3	16i	**32**	15i	**33**	**14**	**33**	18i	**34**	15i	**35**	15i	**37**	15i	**37**	15i	**36**	15i	15i	15i	**42**
CNBP1	[TG]n [TCTG]n [CCTG]n	3q21.3	156	**186**	146	**190**	**122**	**198**	154	**202**	134	**206**	140	**208**	142	**208**	142	**204**	132	136	136	**226**
rs1871922	C or A	3q21.3	A/C		A/C		C	C	A	C	A	**C**	A/C		A/C		A/C		C	C	C	C

Supplementary Table S3: Time dependent somatic instability/stability of the uninterrupted *CNBP* alleles and 17 other microsatellite loci (*DMPK* and the loci included in the new European Standard Set (ESS) of loci, plus SE33) in selected family members. Uninterrupted alleles are shown in bold.Results of the gender specific amelogenin and the rs1871922 genotypes are also shown. For the *CNBP1* locus uninterrupted alleles are shown in bold and are underlined, while "i" indicates interrupted alleles.

Locus	Repeat motif	Chrom. position	Family_2 Index case (26yrs)		Index case (43yrs)		Family_4 Sister of index case (24 yrs)		Sister of index case (40 yrs)	
AMEL (X/Y)	-	Xp22.1-22.3 Yp11.2	X	Y	X	Y	X	X	X	X
TH01 (TC11)	[TCAT]n	11p15.5	8	9.3	8	9.3	6	9.1	6	9.1
D3S1358	TCTA [TCTG]n [TCTA]n	3p25.3	17	18	17	18	15	17	15	17
vWA	TCTA [TCTG]n [TCTA]n	12p13.31	17	17	17	17	18	18	18	18
D21S11	[TCTA]n [TCTG]n [TCTA]n TA [TCTA]n TCA [TCTA]n TCCATA [TCTA]n	21q21.1	29	29	29	29	29	32	29	32
D16S539	[GATA]n	16q24.1	11	14	11	14	11	14	11	14
D1S1656	[TAGA]n [TGA][TAGA][TAGG]n[TG]n	1q42	12	18.3	12	18.3	16	17.3	16	17.3
D19S433	AAGG [AAAG] AAGG TAGG [AAGG]n	19q12	16.2	19	16.2	19	14	14	14	14
SE33 (*ACTBP2*)	[AAAG]n AA [AAAG]n	6q14.2	24.2	31.2	24.2	31.2	15	17	15	17
D10S1248	[GGAA]n	10q26.3	15	16	15	16	15	16	15	16
D22S1045	[ATT]n ACT [ATT]n	22q12.3	11	11	11	11	11	15	11	15
D12S391	[AGAT]n GAT [AGAT]n [AGAC]n AGAT	12p13.2	18	19	18	19	22	22	22	22
D8S1179	[TCTA]n	8q23.1-23.2	13	14	13	14	14	14	14	14
D2S1338	[TGCC]n[TTCC]n	2q35	20	25	20	25	18	20	18	20
D2S441	[TCTA]n	2p14	11	15	11	15	10	11	10	11
D18S51	[AGAA]n	18q21.3	14	15	14	15	17	18	17	18
FGA (FIBRA)	[TTTC]n TTTTTTCT [CTTT]n CTCC [TTCC]n	4q28.2	20	24	20	24	23	24	23	24
DMPK	[CTG]n	19q13.3	5	12	5	12	5	13	5	13
CNBP1	[CCTG]n alone	3q21.3	18i	**34**	18i	**34**	15i	**42**	15i	**43**
CNBP1	[TG]n [TCTG]n [CCTG]n	3q21.3	154	**<u>202</u>**	154	**<u>202</u>**	136	**<u>226</u>**	136	**<u>230</u>**
